# What are the factors driving antimicrobial resistance? Perspectives from a public event in London, England

**DOI:** 10.1186/s12879-016-1810-x

**Published:** 2016-09-02

**Authors:** Enrique Castro-Sánchez, Luke S. P. Moore, Fran Husson, Alison H. Holmes

**Affiliations:** 1 National Institute of Health Research Health Protection Research Unit in Healthcare Associated Infection and Antimicrobial Resistance, Imperial College London, Hammersmith Campus, London, W12 0NN UK; 2 Department of Infectious Diseases, Imperial College London, London, UK; 3 Imperial College Healthcare NHS Trust, London, UK

**Keywords:** Antimicrobial resistance, Public awareness, Health literacy

## Abstract

**Background:**

Antimicrobial resistance is driven by multiple factors. Resolving the threat to human and animal health presented by drug-resistant infections remains a societal challenge that demands close collaboration between scientists and citizens. We compared current public views about key contributing factors to antimicrobial resistance with those expressed by experts.

**Methods:**

Overarching factors contributing to antimicrobial resistance were identified following a review of literature. The factors were then described in plain language and attached to ballot boxes at a public engagement event organised by a university. Responses to each factor were counted at the end of the event.

**Results:**

Four hundred five responses were received from 3750 visitors (11 % response rate). Nearly half of responses (192/405, 47 · 4 %) considered the misuse/overuse of antibiotics in humans as the main determinant of antimicrobial resistance. The misuse of antibiotics in animal health obtained 16 · 3 % (66/405) responses. However, the lack of quick tests to diagnose infections received 10/405 votes (2 · 47 %), and the lack of effective vaccines received one vote (0 · 25 %).

**Conclusions:**

The majority of responses ascribed the emergence of drug-resistant infections to the misuse of antibiotics in human and animals. Suboptimal dosing, availability of diagnostics and environmental contamination were considered less influential on the development of antimicrobial resistance. The growing recognition of broader multifaceted drivers of drug resistance by experts is not yet echoed in the public mind.

## Background

Resolving the threat to human and animal health presented by antimicrobial resistance remains a challenge for health care systems across the world [1]. Although an intrinsic characteristic of micro-organisms, the prevalence of clinically relevant antimicrobial resistance (AMR) has been accelerated by the inappropriate use of antimicrobial agents, in turn driven by several factors. Some of those key determinants include suboptimal prescribing and inadequate public adherence to recommended behaviours such as completion of prescribed antibiotic courses [2].

Public behaviours around antimicrobials are shaped by multiple and interlinked factors, including structural components such as access to adequate medical services and medications [3], narratives about the power of antibiotics [4] and social mechanisms. [5] Public perceptions about AMR may also be influenced by the characteristics of the information available [6]. Such information may not be appropriately formatted for a large segment of citizens [7]. Equally, the population may not have necessary skills to make effective use of the information provided [8]. For such reasons, scientific and health policy messages that focus largely on clinical and epidemiological consequences of inappropriate use of antibiotics may fail to engage the attention of the public. Additionally, scientific evidence that is not closely aligned with public perceptions is likely to risk fostering and encouraging a collective cognitive dissonance about the AMR threat [9].

However, there is increasing evidence of gaps in public knowledge and understanding of messages provided by healthcare workers and clinicians related to antimicrobials and AMR [10]. Further, there is still a paucity of data regarding the explanatory models employed by citizens to understand the development, evolution or transfer of resistance.

Following a recent Delphi round with international experts on key determinants of the global burden of AMR [11], we explored the opinions of members of the public about the same topic to determine the concordance between the two groups.

## Methods

### Factor identification and plain language translation

Nine overarching factors were identified as contributing to the global rise in AMR following review of the national and international scientific literature (briefly, PubMed and the Cochrane Database of Systematic Reviews were searched to identify English language primary research papers, systematic reviews, and meta-analyses (Jan 01 1990–Sep 31 2014) relating to (“factor” OR “driver” OR “cause”) AND “antimicrobial resistance”) [11]. Each of these nine factors was then translated into plain language with the aid of three community representatives. These translations were then piloted on 47 members of the public, and further refinements made to the language used (Table 1).

**Table 1:** Factors identified as driving global antimicrobial resistance

Factors	Plain language translation
Human antimicrobial mis-/over-use	Misuse and/or overuse of antibiotics in humans (e.g. not finishing a course of antibiotics, taking antibiotics for viral, rather than bacterial, infections)
Animal antimicrobial mis-/over-use	Misuse and/or overuse of antibiotics in animals (e.g. farming)
Environmental contamination (including sewage and heavy metals)	Waste products from antibiotics entering the environment (through manufacture, sewage and disposal)
Healthcare transmission	Resistant bacteria being passed between people in hospital and other healthcare areas
Sub-optimal rapid diagnostics	A lack of quick, accurate tests to diagnose infections
Sub-optimal preventative medicine/vaccination	A lack of effective vaccines and poor uptake of existing ones
Sub-optimal dosing, including from substandard and falsified medications	Incorrect dosing of antibiotics in humans (e.g. not adjusting dosage for body weight)
Travel	Human travel from one area of the globe to another, spreading resistant bugs
Mass drug administration in human health	Mass drug administration – i.e. the regular giving of antibiotics to a large group of people (e.g. a whole state or country) to treat an infection, regardless of whether individuals are ill or not

### Participant recruitment

The final plain language summaries were then attached to ballot boxes and placed at a dedicated activity stall at the annual public engagement event organised by Imperial College London university in London, United Kingdom (UK). The stall was manned by a researcher and public attendees of all ages who approached the stall were invited to place one token each in the ballot box in that they felt represented the biggest single cause driving global antimicrobial resistance. Tokens in each box were then summed at the end of the festival and apportioned to each factor.

### Comparison with expert opinion

A previous exercise had been undertaken previously with nine experts in antimicrobial resistance who practice in disparate fields, from molecular biology through to translational antimicrobial resistance research, clinical infection practice and veterinary medicine. This panel undertook a two-round Delphi process to similarly rank the perceived contribution of each factor as a cause of global antimicrobial resistance and in addition also ranked the contributory scientific evidence for each factor, and the potential population affected [11]. The results from the public poll related to the relative contribution of factors towards AMR were compared and contrasted to these expert results to explore any divergence.

### Role of the funding source

The research was funded by the National Institute for Health Research Health Protection Research Unit (NIHR HPRU) in Healthcare Associated Infection and Antimicrobial Resistance at Imperial College London in partnership with Public Health England (PHE). However, the study funders had no influence on how the data were collected, analysed, interpreted or presented. The views expressed are those of the author(s) and not necessarily those of the NHS, the NIHR, the Department of Health or Public Health England.

## Results

Around 15,000 members of the public attended the event, of whom approximately 3750 visited the area containing the survey, where 405 responses (11 % response rate) were received (Table 2). Nearly half of responses (192/405, 47 · 4 %) considered the misuse/overuse of antibiotics in humans as the main determinant of antimicrobial resistance. The misuse of antibiotics in animal health obtained 16 · 3 % (66/405) responses, closely followed by the mass administration of antibiotics to large populations to treat endemic infections. On the other hand, the lack of quick, accurate tests to diagnose infections received 10/405 votes (2 · 47 %), and the lack of effective vaccines, or the poor uptake of existing ones, was given one vote (0 · 25 %).

**Table 2:** Responses by attendees to public engagement event

Factors	n/N (%)
Human antimicrobial mis-/over-use	192/405 (47 · 4 %)
Animal antimicrobial mis-/over-use	66/405 (16 · 3 %)
Mass drug administration in human health	57/405 (14 · 07 %)
Healthcare transmission	36/405 (8 · 89 %)
Travel	17/405 (4 · 2 %)
Sub-optimal dosing, including from substandard or falsified medications	15/405 (3 · 7 %)
Environmental contamination (including sewage and heavy metals)	11/405 (2 · 72 %)
Sub-optimal rapid diagnostics	10/405 (2 · 47 %)
Sub-optimal preventative medicine/vaccination	1/405 (0 · 25 %)

**Fig. 1:**
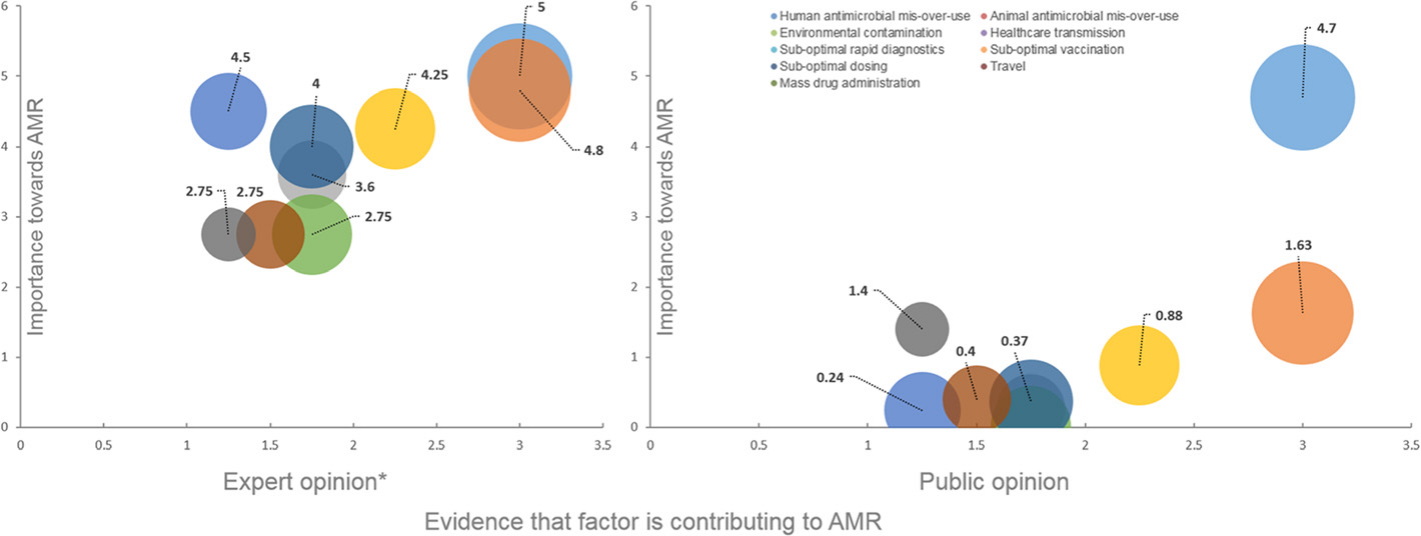
Expert versus public opinion regarding the relative contribution of selected drivers of antimicrobial resistance. Numerical values in graph displaying public opinion results present Tablet 2 percentages normalised to 0–10. For comparison, X-axis and size of bubbles in graph displaying public opinion results use evidence parameters by Holmes, Moore et al. [11]

In general, and with the exception of ‘the misuse of antibiotics in humans, the public attributed less relative importance to each factor than the experts (Fig. 1). The greatest discrepancies in opinion were seen on the significance of lack of diagnostics (considered to be a high driver of AMR by the experts, but of moderate-low importance for the public), and the prominence of suboptimal dosing, ranked within the high-moderate category by the experts, but as moderate-low by the public.

## Discussion

Our survey asked the public attending a science communication event to identify the main reason underlying antimicrobial resistance in the world. Understanding the beliefs and explanatory frameworks used by citizens to make sense about the connections between antimicrobials, microorganisms and hosts, as well as the phenomenon of antimicrobial resistance, can facilitate the design and implementation of public awareness campaigns and strengthen future behaviour change interventions.

Nearly 2/3 of responses ascribed the emergence of resistance to the misuse or overuse of antibiotics in human and animal health. In view of the widely reported prevalence of suboptimal antimicrobial prescribing in human healthcare and its impact on AMR [12], and the evidence suggesting that resistance is intimately associated with antimicrobial use [13], such responses from the public could be considered adequate. Of the two factors, a much larger proportion of individuals considered human health as chiefly responsible for antimicrobial resistance, when compared with animal health. This perspective is remarkable in light of the relative consumption of antimicrobials in veterinary medicine and food production, several orders of magnitude higher than human health [14].

A final group of determinants including travel, suboptimal dosing, environmental contamination and suboptimal rapid diagnostics was considered by respondents to have a lower influence on the development of AMR. The crucial role of rapid diagnostics in AMR seemed clearly undervalued by the attendees to our event. Modern transport routes have proven to be very efficient adjunct mechanisms of pathogen transmission [15]. However, worldwide transfer of antimicrobial resistant organisms does not seem to match the mobility of humans across the globe [16]. Suboptimal dosing across human and animal health remains a persistent challenge, either from insufficient evidence about the optimal management for particular groups such as obese individuals [17], or from substandard medications [18]. Finally, the presence of antimicrobials and antimicrobial-resistant organisms in human food and animal feed as well as the environment and its impact on human and animal health have received growing attention [19]. Our participants attributed to the mass drug administration of antibiotics for public health reasons a moderate role in the growth of resistance. However, the evidence to support such perception is more uncertain [20–22].

The adequate responses we obtained need to be appraised within the UK context, where several awareness campaigns and interventions including public education have been conducted [23]. The effectiveness of such campaigns appears to be conflicting [24–26]. The UK Chief Medical Officer (CMO) has also generated considerable attention to the topic of antimicrobial resistance, developing public engagement materials and effectively guiding the social debate about antimicrobials and resistance in social media [27].

The limited public understanding of the scientific discourse related to antimicrobials may offer an explanation for the uncertain public campaign impact reported [28]. Such narrow understanding may reflect, overall, a lack of scientific skills and health literacy in particular of the population [29, 30]. Unless such deficits are taken into account and remedied, it is likely that recent recommendations directing healthcare workers to educate citizens about antibiotics at the point of clinical care [31] will have limited success.

### Limitations

We conducted our investigation at a public engagement science event aimed at the public organised by a central London university. The ballot boxes were displayed within an area devoted to antimicrobial resistance, so the visitors may have been a self-selected sample of individuals interested in this field, possibly with increased education and information about AMR and its determinants or encouraged to look up information related to antimicrobials in advance. The ballot boxes were not masked so some respondents may have been persuaded to opt for the option which already had the highest number of votes. Equally, we cannot be sure that some responses were not provided by human or animal healthcare workers, scientists or researchers, therefore skewing the proportion of answers for the selected options.

Our activity only allowed people to select the main contributing factor to antimicrobial resistance in their opinion, where in reality antimicrobial resistance is an intricate and ‘wicked’ problem [32]. We did not include an option acknowledging resistance as a natural and evolutionary response of microorganisms to the pressure exercised by antimicrobials, therefore being a phenomenon that could be slowed but not completely stopped.

Due to the design of our activity, we did not have an opportunity to explore social and demographic attributes of those taking part in the survey, which would have been valuable to enrich hypothesis about current explanatory and decision making frameworks used by members of the public. Our findings must therefore be corroborated by surveys with larger samples. We were not able to explore participants’ views about the drivers of such overuse or misuse of antibiotics using qualitative approaches. However, these aspects have been partially described already [33].

## Conclusions

In our study public opinion was largely in agreement with expert opinion characterising human antimicrobial misuse as the greatest contributing factor toward antimicrobial resistance. However, to further progress the public understanding of additional drivers of drug-resistant infections, clear messages will need to be conceptualised, developed and appropriately disseminated, mindful of health literacy issues. As seen with some disease outbreaks, messages supported by facts alone may be insufficient to engage with all population segments, and emotional or practical components may prove more useful content to inform the public and initiate wide ranging discussions on these other issues. In doing so, not only publically funded research streams may be better targeted [34] but also public perception of AMR and its causes might be better informed.

## Abbreviations

AMR: Antimicrobial resistance
CMO: Chief Medical Officer
NIHR HPRU: National Institute for Health Research Health Protection Research Unit
PHE: Public Health England
UK: United Kingdom

## References

[1] Laxminarayan R, Matsoso P, Pant S, et al. Access to effective antimicrobials: a worldwide challenge. Lancet. 2015. doi:10.1016/S0140-6736(15)00474-2. 10.1016/S0140-6736(15)00474-2 26603918

[2] Dar OA, Hasan R, Schlundt J, et al. Exploring the evidence base for national and regional policy interventions to combat resistance. Lancet. 2015. doi: 10.1016/S0140-6736(15)00520-6. 10.1016/S0140-6736(15)00520-6 26603921

[3] Planta MB (2007). The role of poverty in antimicrobial resistance. J Am Board Fam Med.

[4] Hawkings NJ, Butler CC, Wood F (2008). Antibiotics in the community: a typology of user behaviours. Patient Educ Couns.

[5] Smith RA, Quesnell M, Glick L, Hackman N, M’Ikanatha NM (2015). Preparing for antibiotic resistance campaigns: a person-centered approach to audience segmentation. J Health Commun.

[6] Francis NA, Butler CC, Hood K, Simpson S, Wood F, Nuttall J (2009). Effect of using an interactive booklet about childhood respiratory tract infections in primary care consultations on reconsulting and antibiotic prescribing: a cluster randomised controlled trial. BMJ.

[7] de Bont EG, Alink M, Falkenberg FC, Dinant GJ, Cals JW (2015). Patient information leaflets to reduce antibiotic use and reconsultation rates in general practice: a systematic review. BMJ Open.

[8] Public Health England (2015). Local action on health inequalities Improving health literacy to reduce health inequalities.

[9] Rubina GJ, Finna Y, Potts HW, Michie S. Who is sceptical about emerging public health threats? Results from 39 national surveys in the United Kingdom. Public Health. 2015. doi:10.1016/j.puhe.2015.09.004. 10.1016/j.puhe.2015.09.004 PMC4684148 26603602

[10] WHO (2015). Antibiotic resistance: multi-country public awareness survey.

[11] Holmes AH, Moore LS, Sundsfjord A, et al. Understanding the mechanisms and drivers of antimicrobial resistance. Lancet. 2015. doi:10.1016/S0140-6736(15)00473-0. 10.1016/S0140-6736(15)00473-0 26603922

[12] Goossens H, Ferech M, Vander Stichele R (2005). Outpatient antibiotic use in Europe and association with resistance: a cross-national database study. Lancet.

[13] Zhang YB, Li Y, Sun XL (2011). Antibiotic resistance of bacteria isolated from shrimp hatcheries and cultural ponds on Donghai Island, China. Mar Pollut Bull.

[14] Food and Drug Administration (2014). 2011 Summary report on antimicrobials sold or distributed for use in food-producing animals.

[15] Kumarasamy KK, Toleman MA, Walsh TR (2010). Emergence of a new antibiotic resistance mechanism in India, Pakistan, and the UK: a molecular, biological, and epidemiological study. Lancet Infect Dis.

[16] Bedford T, Riley S, Barr IG (2015). Global circulation patterns of seasonal influenza viruses vary with antigenic drift. Nature.

[17] Pai MP (2015). Treatment of bacterial infections in obese adult patients: how to appropriately manage antimicrobial dosage. Curr Opin Pharmacol.

[18] Newton PN, Green MD, Fernández FM, Day NPJ, White NJ (2006). Counterfeit anti-infective drugs. Lancet Infect Dis.

[19] Done HY, Halden RU (2015). Reconnaissance of 47 antibiotics and associated microbial risks in seafood sold in the United States. J Hazard Mater.

[20] Mitjà O, Houinei W, Moses P (2015). Mass treatment with single-dose azithromycin for yaws. N Engl J Med.

[21] West SK, Moncada J, Munoz B (2014). Is there evidence for resistance of ocular Chlamydia trachomatis to azithromycin after mass treatment for trachoma control?. J Infect Dis.

[22] Coles CL, Mabula K, Seidman JC (2013). Mass distribution of azithromycin for trachoma control is associated with increased risk of azithromycin-resistant Streptococcus pneumoniae carriage in young children 6 months after treatment. Clin Infect Dis.

[23] McNulty CAM, Boyle P, Nichols T (2007). The public’s attitudes to and compliance with antibiotics. J Antimicrob Chemother.

[24] WHO (2015). Worldwide country situation analysis: response to antimicrobial resistance.

[25] Lambert MF, Masters GA, Brent SL (2007). Can mass media campaigns change antimicrobial prescribing? A regional evaluation study. J Antimicrob Chemother.

[26] Huttner B, Goossens H, Verheij T (2010). on behalf of the CHAMP consortium. Characteristics and outcomes of public campaigns aimed at improving the use of antibiotics in outpatients in high-income countries. Lancet Infect Dis.

[27] Dyar OJ, Castro-Sánchez E, Holmes AH (2014). What makes people talk about antibiotics on social media? A retrospective analysis of Twitter use. J Antimicrob Chemother.

[28] Wellcome Trust (2015). Exploring the consumer perspective on antimicrobial resistance.

[29] Sørensen K, Pelikan JM, Röthlin F, et al. Health literacy in Europe: comparative results of the European health literacy survey (HLS-EU). Eur J Public Health. 2015;25(6):1053–8. doi:10.1093/eurpub/ckv043. Epub 2015 Apr 5. 10.1093/eurpub/ckv043 PMC4668324 25843827

[30] Castro-Sánchez E, Chang PW, Vila-Candel R, Escobedo AA, Holmes AH. Health literacy and infectious diseases: Why does it matter? Int J Infect Dis. 2016. doi:10.1016/j.ijid.2015.12.019. 10.1016/j.ijid.2015.12.019 26751238

[31] National Institute for Health and Care Excellence (2015). Antimicrobial stewardship: systems and processes for effective antimicrobial medicine use.

[32] Jasper Littmann J, Viens AM. The ethical significance of antimicrobial resistance. Public Health Ethics. 2015. doi:10.1093/phe/phv025. 10.1093/phe/phv025 PMC4638062 26566395

[33] EC Directorate-General for Health and Consumers, Special Eurobarometer 407 (2013). Antimicrobial resistance.

[34] Fitchett JR, Head MG, Cooke MK, Wurie FB, Atun R (2014). Funding infectious disease research: a systematic analysis of UK research investments by funders 1997–2010. PLoS One.

